# Supplementary material for: Differences in Toxic Response Induced by Three Variants of the Diarrheic Shellfish Poisoning Phycotoxins in Human Intestinal Epithelial Caco-2 Cells
Source: Toxins (Basel). 2020 Dec 8;12(12):783. doi: 10.3390/toxins12120783 (PMC7764622; doi:10.3390/toxins12120783)
Supplement: Supplementary file 1 [file toxins-12-00783-s001.pdf]

Supplementary Materials: Differences in Toxic Response induced by three Variants of the Diarrheic Shellfish Poisoning Phycotoxins in Human Intestinal Epithelial Caco-2 Cells

Antoine Huguet, Olivia Drapeau, Fanny Rousselet, Hélène Quenault and Valérie Fessard

Table S1. Target genes and oligonucleotide forward (F) and reverse (R) primers used in this study.

Gene symbol	Accession number	Amplicon length (bp)	Sequence 5' to 3'	Annealing T (C°)
gapdh	NM_002046.5	158	F: GTCAAGGCTGAGAACGGGAA	60
			R: AAATGAGCCCCAGCCTTCTC	
rfc1	NM_002913.4	160	F: TGATGTTGCCCGGAAAGTGT	60
			R: TTTCATGTCACCCCCTGCTG	
rfc4	NM_002916.3	131	F: TGGGCCTGAACTTTTCCGATT	60
			R: GCTTCCCATCTGAGCGACTT	
rpa1	NM_002945.3	159	F: AATGGAAGCTCGGGAATGGG	60
			R: GGTCCACTTGGACTGGTAAGG	
rpa3	NM_002947.4	140	F: GGCATGCTAGCTCAATTCATCG	60.5
			R: TCATCAAGGGGTTCCATCAACTC	
cbl	NM_005188.3	83	F: GGGAAGGCTTCTATTTGTTTCCTG	60
			R: ATGGTCTTGGGGAGTTGGTTC	
grb2	NM_002086.4	71	F: CAAAGCTACTGCAGACGACG	59.5
			R: CACATTCTTCGTTCAAAACCTTGAG	
map2k1	NM_002755.3	79	F: TCAAGTCCTGAAGAAAGCTGGAA	60
			R: TGTCAGGCCTTTTATTACAGCAATG	

mapk8	NM_001278548.1	132	F: TCATGAGCAGAAGCAAGCGT	60.5
			R: AAGCTGCGCATACTATTCCTTGA	
nras	NM_002524.4	71	F: CCACGAACTGGCCAAGAGTTA	60
			R: CTTCAACACCCTGTCTGGTCTT	
rela	NM_021975.3	75	F: CGGCCATGGACGAACTGT	60
			R: TGATCTCCACATAGGGGCCA	
cdkn1b	NM_004064.4	73	F: CAACCGACGATTCTTCTACTCAAAA	60
			R: TTTGGGGAACCGTCTGAAACA	
hras	NM_005343.3	157	F: AGTACAGGGAGCAGATCAAACG	60.5
			R: TTGGCCGAGGTCTCGATGTA	
tgfbr1	NM_004612.3	174	F: CTGGGAAATTGCTCGACGATG	60
			R: ACTCTCAAGGCTTCACAGCTC	
ccnd1	NM_053056.2	157	F: GATGCCAACCTCCTCAACGA	60
			R: GTTCCTCGCAGACCTCCAG	
ccnd2	NM_001759.3	70	F: CTGTCTCTGATCCGCAAGCA	60
			R: ACATGGCAAACTTAAAGTCGGTG	

Table S2. Biological processes, cellular components, and molecular functions related to specific up- and down-regulated genes in Caco-2 cells after 24 hours of exposure to OA, DTX-1, and DTX-2.

**Biological processes**	**Cellular components**	**Molecular functions**
Specifically up-regulated by DTX-1		
regulation of biological process	nuclear lumen	GTPase activity
regulation of cellular process	nucleus	GTP binding
regulation of macromolecule metabolic process	nuclear part	guanyl nucleotide binding
regulation of cellular macromolecule biosynthetic process	intracellular	guanyl ribonucleotide binding
regulation of cellular metabolic process	nucleosome part	transcription regulator activity
regulation of metabolic process	intracellular organelle lumen	
regulation of nucleobase nucleoside nucleotide and nucleic acid metabolic process	intracellular part	
regulation of nitrogen compound metabolic process	organelle lumen	
regulation of macromolecule biosynthetic process	nucleoplasm	
regulation of primary metabolic process		
regulation of gene expression		
regulation of cellular biosynthetic process		
biological regulation		
regulation of biosynthetic process		
regulation of transcription		
transcription		
macromolecule metabolic process		
cellular macromolecule metabolic process		
transcription from RNA polymerase II promotor		
macromolecule biosynthetic process		
patterning of blood vessels		
ossification		
cellular macromolecule biosynthetic process		
transcription DNA dependent		
RNA biosynthetic process		
Specifically up-regulated by DTX-2		
regulation of the force of heart contraction		
signalling pathway		
cell migration		
response to wounding		
cell motility		
localisation of cell		
cellular component movement		

Specifically down-regulated by OA	
response to nutrients	
circulatory system process	
blood circulation	
response to nutrients levels	
response to vitamin	
response to vitamin D	
response to extracellular stimulus	
vitamin metabolic process	
epithelium development	
regulation of epithelial cell differentiation	
epithelial cell differentiation	
response to metal ion	
response to external stimulus	
regulation of cell proliferation	
gland development	
response to retinoic acid	
Specifically down-regulated by DTX-1	
protein-DNA complex subunit organisation	protein-DNA complex
nucleosome organisation	chromosomal part
DNA strand elongation	chromatin
chromatin assembly	nucleosome
protein-DNA complex assembly	nuclear chromosome part
nucleosome assembly	
chromatin assembly or disassembly	
cofactor metabolic process	
DNA conformation change	
DNA packaging	
DNA strand elongation involved in DNA replication	
serine family amino acid biosynthetic process	
chromosome organisation	

Specifically down-regulated by DTX-2		
	membrane fraction<br>insoluble fraction<br>cell fraction<br>mitochondrial tricarboxylic acid cycle enzyme complex	protein heterodimerisation activity

The genes showing specific up or down-regulation were annotated within biological processes, cellular components and molecular functions using GoMiner software. The GO terms had an enrichment score above 1 and a false discovery rate (FDR) score below 0.05.

Table S3. Terms for specific up- and down-regulated genes in Caco-2 cells after 24 hours of exposure to OA, DTX-1, and DTX-2.

Term	Number of associated molecules
Specifically up-regulated by OA	
jak-STAT signalling pathway	3
Specifically up-regulated by DTX-1	
pathways in cancer	23
gap junction	10
long-term depression	8
MAPK signalling pathway	18
pathogenic *Escherichia coli* infection	7
chronic myeloid leukaemia	8
ubiquitin-mediated proteolysis	11
leukocyte transendothelial migration	10
neurotrophin signalling pathway	10
pancreatic cancer	7
natural killer cell-mediated cytotoxicity	10
jak-STAT signalling pathway	11
B cell receptor signalling pathway	7
Specifically up-regulated by DTX-2	
pathways in cancer	8
chronic myeloid leukaemia	4
erbB signalling pathway	4
Specifically down-regulated by DTX-1	
mismatch repair	6
DNA replication	6
nucleotide excision repair	5

The genes showing specific up- and down-regulation for each toxin were annotated within terms using the DAVID Functional Annotation Tool associated with KEGG pathways. The terms had p-values below 0.05.
